# Liberty of Science in Germany

**Published:** 1895-09

**Authors:** 


### LIBERTY OF SCIENCE IN GERMANY.

[Translated from the manuscript by B. Fincke, M. D., Brooklyn, N. Y.]

The following experience of a medical student in Germany is not without its meaning even in our Republic, inasmuch as it gives a solemn warning against the growing despotism of the allopathic authorities. The revolutionary tendency of the medical faculties in Germany shows that the powers-that-be stop at no means, as low as they may be, to disregard the laws of the State and common decency when the unfortunate student who is at their mercy, applies for examination in order to receive his diploma, and with it the right of practicing the healing art, if he leans to Homœopathy. May he be ever so proficient in his studies and worthy of the distinction which the desired diploma is to confer upon him, they shake their brutal fist in his face, and he must bow down before them if they have detected upon him the odor of the homœopathic heresy. Then, after they have forced him to the indignity of recanting, they deny him flatly the right given him by the law, and send him his way.

This is liberty of science with a vengeance! It is rather the liberty which the ruling medical school is taking with the law of the land and those abiding by the law.

The author tried in vain to publish his manuscript in the German papers till the faithful lay-paper, *Homœopathische Monatsblätter*, edited by Mr. A. Zœppritz, in Stuttgart, brought it out. I received the manuscript from Dr. C. Kunkel in Kiel, and thought it well to publish it in our journal, in order to show those, who think nothing so good except it comes from Europe, how much they are in error.

### Liberty of Science in Germany.

Originally intended and prepared for a minister, I resolved to devote myself to medicine, after having experienced on my own body the superiority of the homœopathic healing method over the common medicines. My grandfather having been a physician of the allopathic school, was the reason why my family always prevented me from turning my attention to the homœopathic healing art. Since, at this moment, no professorship for Homœopathy exists at the universities of Germany, I applied to a prominent representative of this school, with the request to give me instruction and proper direction. At the same time I carried on my studies at the University like every other medical student. After the preliminary medical examination in Kiel, I visited the clinics of the University of Berlin, and at the same time the polyclinic of the Society of the Homœopathic Physicians of Berlin, so that I could compare daily both methods of healing with their therapeutics and results. After returning to Kiel my teacher, mentioned above, made me his stationary assistant, because on account of his advanced age he could no more attend to his extended practice alone. From many sides I was warned and urged to leave the homœopathic healing method for my own good. Convinced of its correctness, however, I did not submit to the temptation, and continued to study assiduously at home and at the sick bed. I had need of my time, for the study of the homœopathic materia medica needs years for any one who means it earnestly.

For the sake of further improvement I assisted several homœopathic physicians, especially one nearly related to me, in the summer of 1892, during the cholera epidemic in Hamburg, on which I was incautious enough to publish a small paper. Later I left Kiel under the pretext of traveling for several months. I thought I should do so on account of the warnings of friends to keep my abode as secret as possible. In truth, however, I reported myself for the examination of a larger university of Germany, hitherto unknown to me, viz.: Leipzig.

I gave satisfaction on every point, partly with the highest approbation. For the last object I had selected the examination in hygiene. For that purpose I presented myself to the examinator, but the examination was deferred several times without my knowing any reason for the delay. Finally the time was appointed, and I was examined very thoroughly. At the close, when I was talking about the cholera, among others I

had drawn the theme: Preventive measures against contagious diseases. The examining gentleman suddenly said: "You have, in 1892, in the *Nachrichten von Husum* (Husum is my home), written about cholera. Do you remember what protective remedy you recommended in that paper?" I had named as a protective means Hering's Sulphur-milk, and for remedies Veratrum, Cuprum, and Arsenicum, recommended also by Professor Hugo Schultz in Greifswald, and as analepticum Camphor. Now at this point a flood of invectives was poured over me, starting with the assertion that all that was swindle and nonsense, etc. Finally the examinator expressed himself with regret: "*That a circular of the medical faculty of Kiel had been sent to all the medical faculties of Germany with that article on cholera as evidence.*" He said he had it in his power to let me fall through, but after the impression which he had received from me, he did not want to do so. He was honest, but still he could give me no certificate (Zensur). If the matter had come from an unknown informer he would have thrown that circular into the waste-basket, but under the circumstances he could not do so, out of respect for the faculty. At last he gave me the following advice: I should write a letter to the faculty in Kiel that I regretted to have published that paper on cholera, and that I had proved by my examination that I was able to do justice to the conception of modern science, etc. If then I would bring to the examinator the receipt of this letter by the faculty in Kiel, he would give me my certificate. I wrote immediately, but in spite of my begging for a receipt by return mail, I received no answer from the dean. I was the more excited, as I wanted to do my military duty for my year of service as a voluntary physician. After a series of days I again went to my examinator. He was very amiable, excused himself on account of the expressions he had used in the excitement of the moment, took pity upon me, and regretted that he could not act otherwise on account of the faculty. Then under his eyes and with his assistance he made me write another letter, somewhat more extensive and polite than I had done. The letter was registered and receipted by the post-office. Though the matter was denoted to be urgent, no answer of the dean arrived. In consequence of the continued excitement I became sick. A dear homœopathic colleague interested himself very much in my behalf, and comforted me as well as he could. I informed my examinator, who sent his assistant, and by order of his chief caused me to write to Kiel for a third time in the same manner as the second time. As soon as this letter was brought to the examinator he wrote in my certificate (Zensur) the mark "good," and delivered it to the proper authority. Only two days later came a short note to me from Kiel, in which the receipt of the first two letters was acknowledged. I handed it immediately to the examinator, and after a short time received my approbation by the government.

During my leisure hours I had, at the advice of the director of the female clinic of the University, where I went through the examination, elaborated a dissertation and handed it to the dean of the faculty. The dean was very reserved and opined that I should get my diploma at the University from which the circular had been emitted. In spite of this I presented to him my work and with it the graduation dues. After a few weeks I received back my dissertation and my money with a decree of repulse.

That I could never graduate in Kiel was, according to my opinion, clearly discernible by the fact that the faculty in Kiel had persecuted me, so to say, like a criminal. Besides, shortly after, the following event happened in Kiel: A physician at this place who, besides other methods, had also used the homœopathic one in his practice—he was by no means a strict or one-sided homœopath—wanted to graduate at the faculty there, hardly two years after he had gone through the State examination and the *examen vigorosum*. At that time he could not have graduated because the faculty had been apprised that he was a homœopath, as he thinks, by the same gentleman who also showed his ill-will against me. Now he intimated to him that before all he must publicly forswear Homœopathy. This physician therefore—incomprehensible to me—declared publicly, in the *Kieler Zeitung*, that he had given up his homœopathic practice and was studying for a specialty. This declaration he sent to the faculty, and what did this faculty do now? After long deliberation it refused him for fear that a "relapse" might take place. Thus he was cheated out of his rights.

After long consideration I left Kiel, and followed friendly advice to hand my dissertation in to the medical faculty of the University of old renown, Jena. Though I was a perfect stranger there it was accepted by the faculty. I stood the examination for graduation well and received my diploma as soon as my dissertation had appeared in print.

I pass over sundry things and only want to add that later on I went through the Royal Board of Examination in Berlin successfully, for the right of self-dispensation of homœopathic medicines. Now, consider: *I was persecuted by a Prussian faculty for inclination to a healing method which is recognized as such by the Prussian State through a Royal board of examination.*

What afterward I had to encounter, on account of my always open adherence to the homœopathic healing method I do not publish for the present. Against the advice of my friends I have forborne to apply to the courts.

Dr. W., in K.

